# Ceramic soft tissue trimming bur gingival depigmentation: clinical performance and patient experience. A split mouth randomized controlled trial

**DOI:** 10.1186/s12903-024-04345-z

**Published:** 2024-05-23

**Authors:** Sally K Nassar, Hala Ahmed Abuel-Ela, Yasmine Ahmed Fouad

**Affiliations:** 1 Department of Oral Medicine, Periodontology, and Oral Diagnosis, Faculty of Dentistry, Ain Shams University, Organization of African Unity St, El Qobba Bridge, Cairo, Egypt; 2 Department of Oral Medicine, Periodontology and Oral Diagnosis, Faculty of Dentistry, Ain Shams University and Misr International University, Cairo, Egypt

**Keywords:** Gingiva, Gingivoplasty, Pigmentation, Hyperpigmentation, Melanin, Dental esthetics, Cosmetic dentistry

## Abstract

**Background:**

The ceramic soft tissue trimming bur (CeraTip™) was initially introduced for use in gingivoplasty but has recently been used for gingival depigmentation. The aim of this study is to compare the efficacy of depigmentation between the novel CeraTip™ and the gold-standard surgical scalpel technique.

**Methods:**

Eight healthy, nonsmokers with moderate to severe gingival hyperpigmentation in both arches were randomly assigned for CeraTip™ depigmentation in one arch as the test group (TG) and scalpel depigmentation in the opposite arch as the control group (CG). Pigmentation indices were used to assess clinical performance. Treatment time, pain level, and esthetic satisfaction were the parameters of patient experience. The assessments were performed at baseline, one week, one month, and three months.

**Results:**

At all assessment visits, pigmentation intensity represented by the Dummet oral pigmentation index (DOPI), and pigmentation distribution represented by the Hedin melanin index (MI), were significantly lower than those at baseline (*p* < 0.001) in both groups. When comparing the two groups, Scalpel depigmentation had better initial clinical outcomes, while CeraTip™ had less visible repigmentation, pain scores, treatment time, and greater esthetic satisfaction. However, none of the differences were statistically significant.

**Conclusion:**

Both techniques successfully removed gingival hyperpigmentation with comparable clinical performance. The patients preferred CeraTip™ depigmentation.

**Trial registration:**

The study protocol was registered on 11/09/2023 on the www.clinicaltrials.gov database (NCT06031116) after the approval of the Ethics Committee, Faculty of Dentistry, Ain Shams University (FDASU-Rec012124).

## Background

Dark gingival tissue or gingival hyperpigmentation compromise the harmony of the smile and overall facial esthetics. Many individuals, especially those with a high smile line, seek treatment for hyperpigmentation [1].

Gingival hyperpigmentation is caused by highly active melanocytes, resulting in excessive deposition of melanin pigments in the basal and suprabasal layers of the epithelium. This increase in melanocyte activity can be physiological, pathological, drug-induced, or caused by smoking. Accordingly, a meticulous medical and personal history should be obtained to determine the etiology of melanin hyperpigmentation [2].

A plethora of techniques, both surgical and nonsurgical, have been utilized to remove melanocytic pigmentation. Surgical techniques include surgical stripping, bur abrasion, cryosurgery, electrosurgery, lasers, or masking of the pigmented gingiva using free gingival autografts and acellular dermal matrix allografts. The nonsurgical techniques include ascorbic acid (vitamin C), salicylic acid, glycolic acid, trichloroacetic acid, and phenols. Many studies have reviewed gingival depigmentation techniques. However, there is no consensus on which technique is the most effective, pleasant, or reliable [3, 4].

The available treatment modalities either involve a bloody surgical field, require sophisticated equipment, or demand multiple applications to achieve the desired outcome [3, 5]. However, there is no consensus on which technique is better regarding clinical outcomes, stability of results, or patient satisfaction.

CeraTip™ [Komet USA (Rock Hill, SC 29,730, USA)] is a flame-shaped cylinder made of ceramic oxide. These tips are inserted in a high-speed handpiece and used without a water coolant. The heat from friction ablates the tissues and simultaneously coagulates the blood vessel ends, resulting in minimal bleeding [6]. Gingival depigmentation by Ceramic soft tissue trimming bur has been reported in a case study [7] and a case series [8] and achieved results comparable to those of laser in a randomized clinical trial [6]. Ceramic soft tissue trimming bur depigmentation is a simple, effective, and minimally invasive clinical procedure [7].

This study aimed to compare gingival depigmentation using a CeraTip™ versus the conventional surgical scalpel technique because both are simple, cost-effective methods for depigmentation.

## Participants and methods

### Study design, patient grouping, and randomization

This study is a prospective split-mouth randomized clinical trial (RCT). The dental arches of each patient were randomly allocated to the two groups at a ratio of 1:1 according to the predetermined computer-generated randomization. The allocation was concealed by the researcher (YF) in sequentially numbered envelopes until the intervention. The participants were randomly assigned for CeraTip™ depigmentation in one arch (**TG**) and scalpel depigmentation in the opposite arch (**CG**).

### Study sample, sample calculation, and power analysis

This study was conducted on sixteen dental arches of eight patients seeking treatment for gingival hyperpigmentation for esthetic reasons. The patients were recruited from the Department of Oral Medicine, Periodontology and Oral Diagnosis outpatient clinic, Faculty of Dentistry, Ain Shams University.

A power analysis was designed to have adequate power to apply a two-sided statistical test of the null hypothesis that no difference would be found between different tested groups. By adopting an alpha (α) level of 0.05 (5%), a beta (β) level of 0.2 (i.e., power = 80%), and an effect size (d) of (1.931) calculated based on the results of a previous study with the same primary outcome [9]; the predicted sample size (n) was a total of 6 cases. The sample size was increased by 25% to compensate for possible drop-out during different follow-up intervals to be a total of 8 patients. Sample size calculation was performed using G*Power version 3.1.9.7^1^.

### Ethics approval

This study was conducted after approval was granted by the Research Ethical Committee at the Faculty of Dentistry—Ain Shams University (FDASU-Rec012124) and was registered on 11/09/2023 at https://clinicaltrials.gov/ under the number (NCT06031116).

### Eligibility criteria

Fifteen patients were assessed to enroll eight subjects who met the eligibility criteria. The inclusion criteria were patients with more than one short continuous ribbon of pigmentation in both arches: ≥ 3 on the Hedin melanin index, good oral hygiene, thick gingival phenotype, and ASA (American Academy of Anesthesiologists) class I individuals.

Subjects were excluded if they were smokers, had periodontally compromised teeth, were pregnant or lactating, had a physical or mental impairment, had pathologies that cause gingival pigmentation (e.g.: Addison’s disease, Albright’s syndrome, Kaposi sarcoma), were taking medication that may induce pigmentation (e.g.: bleomycin, minocycline, chloroquine, quinine).

### Treatment protocol

#### Preoperative preparations and instructions

All patients received nonsurgical periodontal phase I therapy (scaling and polishing) two weeks before the depigmentation session and were instructed to perform proper oral hygiene.

#### TG: ceramic soft tissue trimming bur (Cerabur) depigmentation

**Fig. 1:**
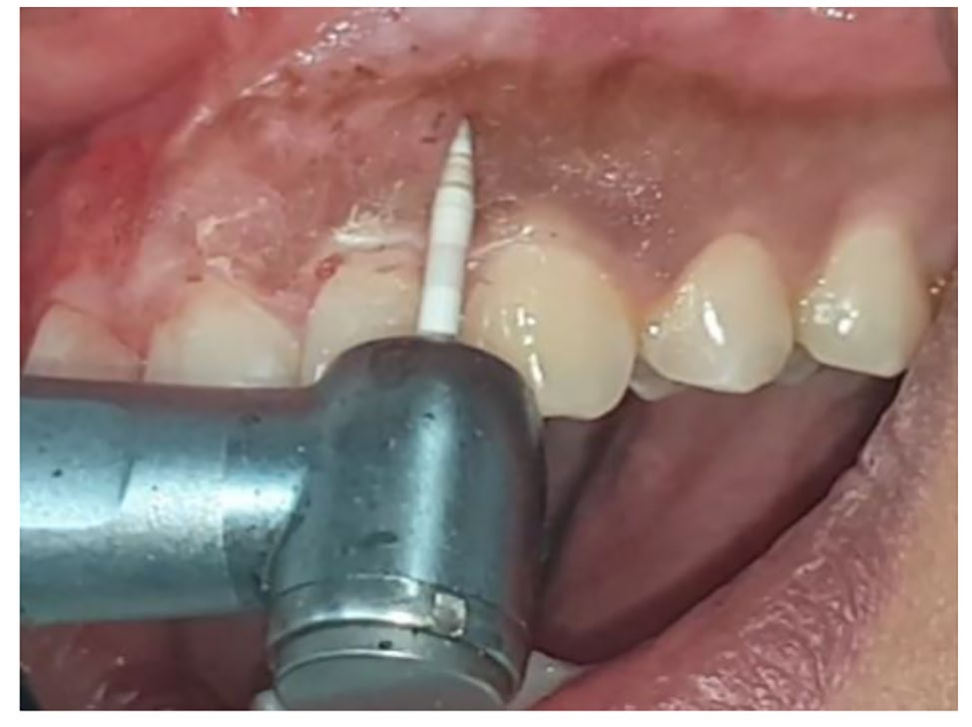
Depigmentation using ceramic bur

The Buccal infiltration technique was used to achieve local anesthesia (articaine with epinephrine 1:100:000, Laboratories Inibsa, Spain). CeraTip™ was used in a high-speed handpiece without a water coolant to remove the epithelial layer and excise and contour the gingival soft tissues. Figure 1. During the procedure, the gingiva and the CeraTip™ were cleaned of gingival debris with a sterile gauze soaked in saline.

#### CG: conventional scalpel surgical scraping technique

Local anesthesia was achieved in the same manner as the TG. The pigmented gingival epithelium was scraped using a no. 15 Bard Parker blade. Care was taken to include the pigmented epithelium at the tip of the interdental papilla and the mucogingival junction. Hemostasis was obtained with sterile gauze and direct pressure.

### Postoperatively

The exposed surface was irrigated with saline, and the surgical area was covered with a periodontal pack (COE-PAK, GC America) for one week for both groups. The patients were instructed to avoid hot and spicy food for 24 h after surgery and to continue mechanical oral hygiene while avoiding the surgical area. Ibuprofen (200 mg) was prescribed immediately after surgery, and the patients were advised to continue with the medication for three days after surgery if pain was experienced.

#### Assessment

**Fig. 2:**
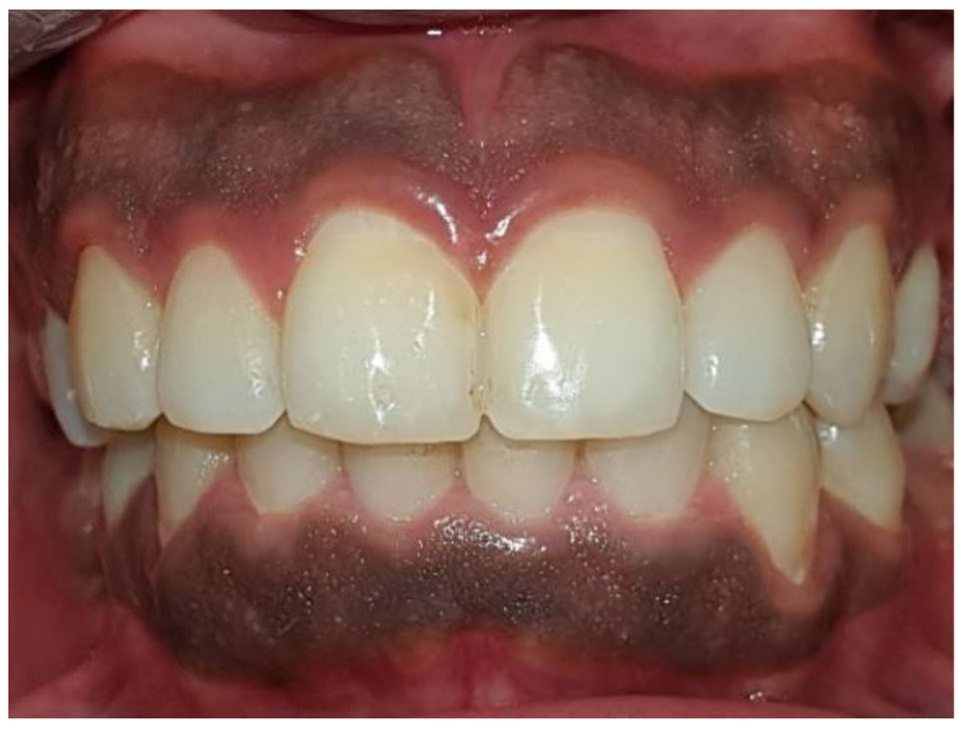
Preoperative picture of a patient with melanin hyperpigmentation in both arches

**Fig. 3:**
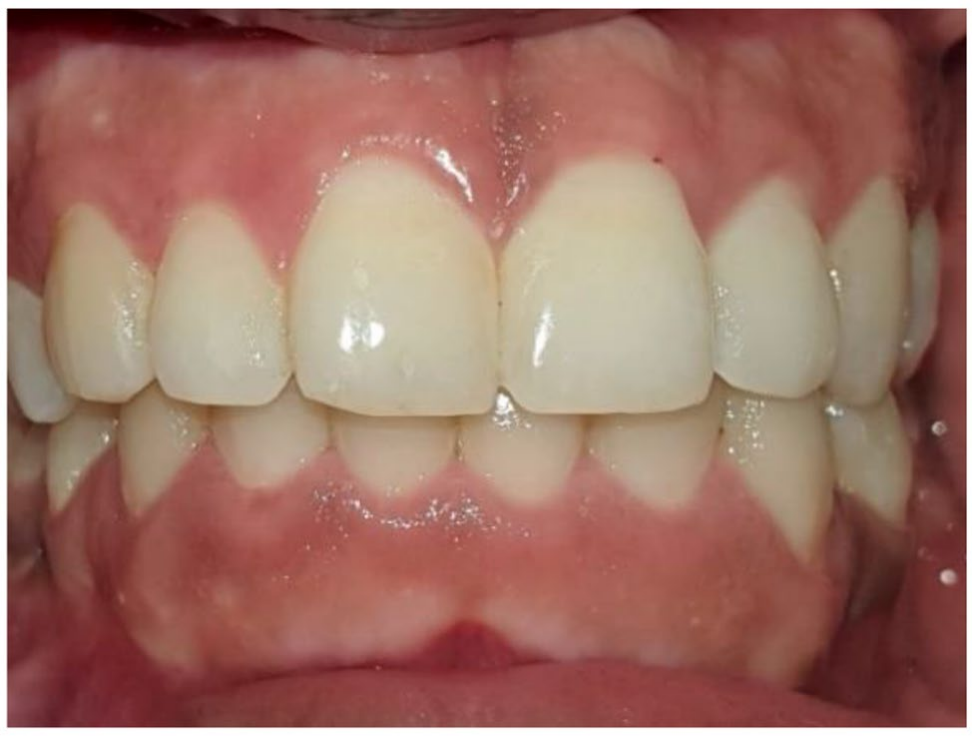
Same patient 3 months postoperatively. Upper arch CeraTip depigmentation, lower arch scalpel depigmentation

Clinical assessment was performed at baseline and after one week, one month, and three months. Figures 2 and 3.

The following parameters were assessed:

#### Pigmentation indices: Dummet oral pigmentation index (DOPI): (Primary outcome)

The *degree* of gingival pigmentation was scored as **0**: pink tissue; **1**: mild light brown tissue; **2**: medium brown or mixed brown and pink tissue; **3**: deep brown/ blue–black tissue.

#### Hedin melanin index (MI)

The *extent* of gingival pigmentation was scored as **0**: no pigmentation; **1**: one or two solitary units of pigmentation in the papillary gingiva; **2**: >3 units of pigmentation in the papillary gingiva without the formation of a continuous ribbon; **3**: >1 short continuous ribbons of pigmentation; **4**: one continuous ribbon including the entire area between canines.

### Parameters of patient experience: (secondary outcomes)

#### Operating time

The time needed to complete each procedure was calculated in minutes using a stopwatch from the time the local anesthetic was applied until the periodontal pack was placed.

**Patient’s esthetic satisfaction** was reported using the **Global Aesthetic Improvement Scale (GAIS)** where **1**: Excellent improvement; **2**: Very improved; **3**: Improved; **4**: unaltered; **5**: worsened. The patients reported their degree of satisfaction with the procedure’s esthetic outcome one month postoperatively.

**Pain perception** was reported using the **visual analog scale (VAS)** at 24 h, 2 days, 3–5 days, and 7 days postoperatively. Zero indicates minimum pain, and ten indicates maximum pain. The patients were asked to keep a diary of the perceived pain levels as well as their analgesic consumption, if any.

##### Data management and statistical analysis

Numerical data were presented as mean and standard deviation values. The data were analyzed for normality using the Shapiro-Wilk test. The nonparametric data were analyzed using Friedman’s test, followed by the Nemenyi post hoc test. The significance level was set at *P* < 0.05 for all tests. Statistical analysis was performed with R statistical analysis software version 4.3.0 for Windows^2^.

**Fig. 4:**
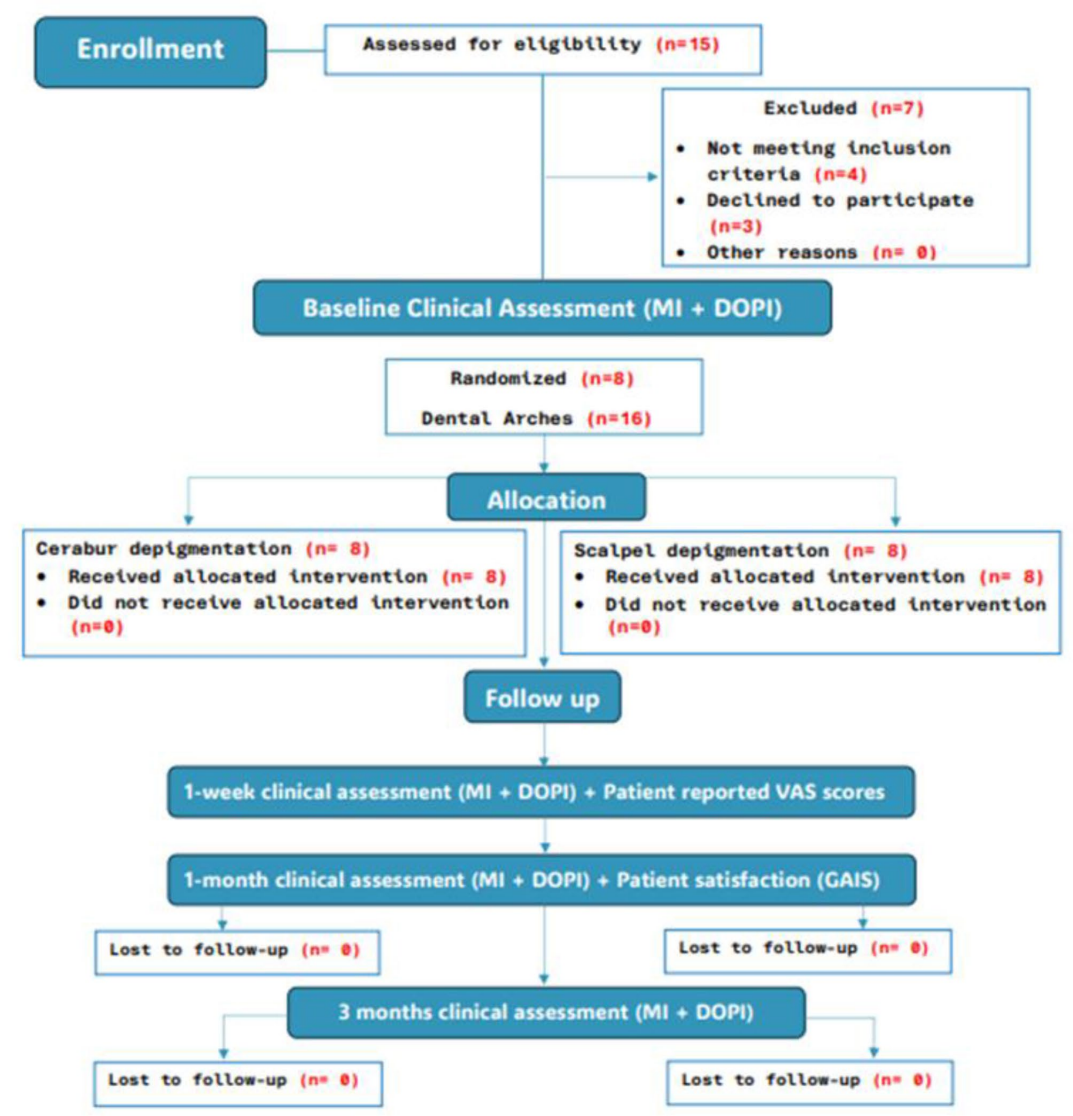
Flowchart of the study illustrating patient recruitments, appointments, and procedures

## Results

Eight patients were enrolled in the study with a mean age of 23.88 ± 2.85 years. The gender distribution was 2 males (25%) and 6 females (75%). All the participants met the eligibility criteria and received treatment and control interventions on either arch, depending on the randomization. Figure 4.

### Clinical outcomes

#### Dummet oral pigmentation index (DOPI)

for *both* the **TG and CG** groups, there was a significant difference between the values measured at different intervals (*p* < 0.001). The highest value was found at baseline (2.25 ± 0.71), followed by three months (0.12 ± 0.35), while the lowest value was found at one month postoperative (0.00 ± 0.00). Post hoc pairwise comparisons showed that the baseline values were significantly greater than those measured at other intervals (*P* < 0.001). Table (1).

**Table 1:** Inter- and intragroup comparisons, and mean and standard deviation (SD) values for DOPI for both groups (values with different superscript letters within the same vertical column are significantly different)

Time	DOPI (Mean ± SD)		*P*-value
	Test	Control	
Baseline	2.25 ± 0.71^a^	2.25 ± 0.71^a^	1
1 month	0.00 ± 0.00^b^	0.00 ± 0.00^b^	1
3 months	0.12 ± 0.35^b^	0.12 ± 0.35^b^	1
*p*-value	< 0.001*	< 0.001*	

* Significant (*p* ≤ 0.05)

#### Hedin melanin index (MI)

The percentage change in MI at different intervals, the **CG** (93.75 ± 17.68) had greater change than the **TG** (89.58 ± 14.60) from baseline to 1 month, and the **TG** (80.21 ± 22.69) had greater change than the **CG** (77.08 ± 28.43) from baseline to 3 months. Both differences were not statistically significant. (*P* = 0.854) from baseline to 1 month and (*P* = 0.812) from baseline to 3 months. Inter- and intra-group comparisons of MI scores are shown in Table (2).

**Table 2:** Inter- and intragroup comparisons, mean and standard deviation (SD) values for MI for both groups at different time intervals (values with different superscript letters within the same vertical column are significantly different)

Time	HEDIN Melanin index (Mean ± SD)		*p*-value
	Test	Control	
Baseline	3.75 ± 0.46^a^	3.75 ± 0.46^a^	1
1 month	0.38 ± 0.52^b^	0.25 ± 0.71^b^	0.850
3 months	0.75 ± 0.89^b^	0.88 ± 1.13^b^	1
*p*-value	< 0.001*	< 0.001*	

* Significant (*p* ≤ 0.05)

#### Patient experience

**Fig. 5:**
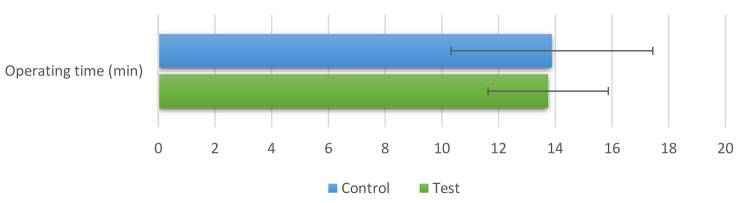
Bar chart showing the mean and standard deviation values (error bars) for both group’s operating time (min)

**The operating time of the CG** (13.88 ± 3.56) was greater than that of the **TG** (13.75 ± 2.12); However, the difference was not statistically significant (*P* = 0.925). Figure 5.

**Fig. 6:**
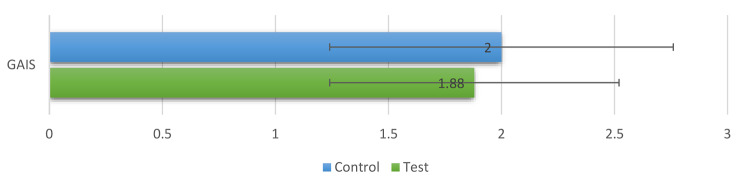
Bar chart showing mean and standard deviation values (error bars) for GAIS for both groups

**Patients’ esthetic satisfaction (GAIS)** was greater in the CG (2.00 ± 0.76) than the TG (1.88 ± 0.64); however, the difference was not statistically significant (*P* = 0.766). Figure 6.

#### Pain perception (VAS)

The CG pain scores were greater than those of the TG on days 1, 2, and 3–5, but the differences were not statistically significant (*p* = 0.169, *p* = 0.752, and *p* = 0.571), respectively. One week postoperatively, only one patient in the **TG** reported residual pain, while no patient reported pain in the **CG**. The difference was not statistically significant (*P* = 1). Table 3.

**Table 3:** Inter- and intragroup comparisons, and mean and standard deviation (SD) values for the VAS score for both groups (values with different superscript letters within the same vertical column are significantly different)

Time	VAS (Mean ± SD)		*p*-value
	Test	Control	
day 1	1.88 ± 2.42^a^	3.38 ± 2.00^a^	0.169
day 2	2.50 ± 2.56^a^	3.12 ± 1.81^a^	0.752
day 3–5	1.25 ± 1.58^a^	1.62 ± 1.19^ab^	0.571
1 week	0.12 ± 0.35^a^	0.00 ± 0.00^b^	1
*p*-value	**0.062**	< 0.001*	

* Significant (*P* ≤ 0.05)

## Discussion

This RCT aimed to compare both techniques regarding clinical outcomes, pain perception, patient satisfaction, and the stability of the results. To the best of our knowledge, there are no published clinical trials comparing the outcomes of gingival depigmentation by ceramic soft tissue trimming bur (CeraTip™) with those of the surgical stripping technique.

The scalpel technique is still the gold standard for gingival depigmentation. The popularity of the scalpel technique is due to its efficiency, affordability, and simplicity. However, this technique has the drawbacks of a bloody field of surgery, probable infections, and a high recurrence rate [10–12]. CeraTip™ was selected for this study because it is a novel and promising technique for gingival depigmentation. It is affordable, easy to use and has the advantages of good hemostasis, and cleanliness of the operating field [7].

The study was conducted in a split-mouth fashion [13, 14]. This design was selected to avoid intersubject variables such as age, facial complexion, genetics, and environmental risk factors. The intersubject variables could induce bias in the estimated treatment effect and the individual’s pain perception [15]. The decision to employ the arch rather than the quadrant design was made to avoid color disparity in the same arch. Carry-over effects may induce bias in split-mouth RCTs [16]. Consequently, in this study, one dental arch of each patient was treated according to the assigned randomization, and the opposing arch was only treated once the patient reported no pain (Zero on the VAS).

Periodontal pack placement is not mandatory. However, there are multiple benefits to applying a periodontal pack postsurgically, including less postoperative pain reported by the patients and slightly better healing [17, 18]. Applying a periodontal pack after both procedures was in accordance with previous studies [6, 7, 12, 19].

The DOPI and the MI were both used to obtain more accurate results, as the DOPI is based on the color intensity of the gingival pigment, whereas the MI is based on the extent and distribution of the pigment. Previous studies have used two pigmentation indices for the same reason [6, 20]. 

The present study’s results showed a statistically significant reduction in DOPI values in both groups from baseline. Gholami et al., 2018, compared surgical stripping and ErCr: YSGG (erbium, chromium-doped yttrium, scandium, gallium, and garnet) laser depigmentation, and Negi et al., 2019, compared ceramic soft tissue trimming bur and diode laser depigmentation. Both studies reported a significant decrease in pigmentation indices from baseline [6, 21].

The percentage change in the MI differed for both groups, with the CeraTip™ having a lower change from baseline to 1 month and a higher change from baseline to 3 months. This shows better initial clinical outcomes of the surgical stripping technique and superior stability of the CeraTip™ results. However, the difference was not statistically significant.

These findings were analogous to those of Gholami et al., 2018, who found no difference between the recurrence rates of scalpel and ErCr: YSGG laser [21]. However, these findings contrast with those of Penmetsa et al., 2019 who reported significantly higher recurrence rates with scalpel depigmentation than with cryosurgery [22].

Gingival repigmentation after depigmentation is one of the drawbacks of this procedure. It is hypothesized that gingival repigmentation occurs due to the migration of neighboring melanocytes. Repigmentation is contingent on many factors including smoking, genetic and environmental influences, and the technique used for depigmentation [19]. After surgical depigmentation, Ginwala et al. reported repigmentation in 50% of the cases after 24 to 55 days, while Perlmutter et al. reported the first signs of repigmentation after 32 months and full repigmentation after seven years [23]. The repigmentation rate of the two techniques was measured at the end of the follow-up period, three months postoperative, to compare the stability of the results [12]. DOPI and MI values were the highest at baseline, while the 1-month values were the lowest. The values increased from the 1-month to the 3-month assessment visit due to the re-appearance of some pigments in the form of spots, dots, or stria in some of the subjects [23].

The VAS was used to evaluate patients’ pain perception in each arch due to its simplicity and reliability [24]. There was no statistically significant difference between patient-reported VAS scores. These results contrast with those of Negi et al., 2019 who reported significantly more pain at sites treated with ceramic soft tissue trimming bur than at site treated with diode laser [6]. The pain levels in this study were comparable to those in previous studies [25, 26] that reported more pain at sites treated with scalpel than at sites treated with laser and aligned with studies [12, 21] that did not find any significant difference in pain levels when comparing lasers with scalpels. The frictional heat of the CeraTip™ caused tissue coagulation and minimal bleeding. The localized heat causes coagulation, protein denaturation, drying, vaporization, and carbonization. As a result, blood vessels and sensory nerve endings are sealed. The frictional heat produced while using the CeraTip™ may explain the slightly lower pain levels in the CeraTip™ sites than in the scalpel sites [6, 27].

The GAIS was adopted from plastic surgery and cosmetic literature [28, 29] to measure the patient’s level of satisfaction with the aesthetic outcome. CeraTip™ had better patient satisfaction scores. More patients appreciated the less invasive and less bloody nature of the procedure over the scalpel procedure.

## Conclusion

The clinical performance of the CeraTip™ and the scalpel techniques was similar, while the overall patient experience (pain, esthetic satisfaction, and treatment time) was more favorable for the CeraTip™ group. CeraTip™ depigmentation is an effective, bloodless, easy-to-perform technique that does not require a sophisticated armamentarium. CeraTip™ depigmentation is a practical substitute for the standard scalpel stripping technique.

### Recommendations

- More clinical trials with larger sample sizes and longer follow-up periods are needed to further evaluate the efficacy, and stability of the ceramic soft tissue trimmer’s results in gingival depigmentation.
- Further research comparing ceramic soft tissue trimming bur with other depigmentation techniques is needed.
- Histological studies should be performed to further understand the mechanism of repigmentation and the time required for pigmentation to return to baseline levels.

## Data Availability

The data supporting the findings of this study are available upon request from the corresponding author.

## Footnotes

1 Faul, Franz, et al. “G* Power 3: A flexible statistical power analysis program for the social, behavioral, and biomedical sciences.” *Behavior research methods* 39.2 (2007): 175–191.

2 R Core Team (2023). R: A language and environment for statistical computing. R Foundation for Statistical Computing, Vienna, Austria. URL https://www.R-project.org/.

## Abbreviations

CeraTip™: Ceramic soft tissue trimming bur
TG: Test group
CG: Control group
DOPI: Dummet oral pigmentation index
MI: Melanin Index
RCT: Randomized controlled trial
ASA: American Academy of Anesthesiologists
GAIS: Global Aesthetic Improvement Scale
VAS: Visual analog scale
ErCr:YSGG: Erbium, chromium-doped yttrium, scandium, gallium, and garnet
